# Supplementary material for: Genomic Characteristics of Feline Anelloviruses Isolated from Domestic Cats in Shanghai, China
Source: Vet Sci. 2023 Jul 7;10(7):444. doi: 10.3390/vetsci10070444 (PMC10385657; doi:10.3390/vetsci10070444)
Supplement: Supplementary file 1 [file vetsci-10-00444-s001.zip › Supplementary Figure S1.pdf]

Supplementary Figure S1.

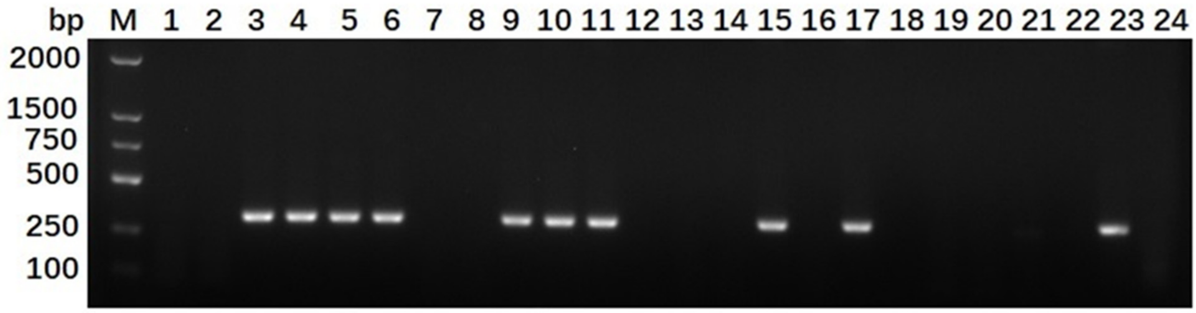

Supplementary Figure S1. Electrophoresis revealed FcTTV detection by PCR method.
